# KLF2 in Regulation of NF-κB-Mediated Immune Cell Function and Inflammation

**DOI:** 10.3390/ijms18112383

**Published:** 2017-11-10

**Authors:** Prerana Jha, Hiranmoy Das

**Affiliations:** Vascular Biology and Stem Cell Research Laboratory, Department of Biomedical Sciences, School of Pharmacy, Texas Tech University Health Sciences Center, Amarillo, TX 79106, USA; prerana.jha@ttuhsc.edu

**Keywords:** KLF2, NF-κB, immune cells, inflammation and diseases

## Abstract

*KLF2* (Kruppel-like factor 2) is a member of the zinc finger transcription factor family, which critically regulates embryonic lung development, function of endothelial cells and maintenance of quiescence in T-cells and monocytes. It is expressed in naïve T-cells and monocytes, however its level of expression decreases during activation and differentiation. KLF2 also plays critical regulatory role in various inflammatory diseases and their pathogenesis. Nuclear factor-kappaB (NF-κB) is an important inducer of inflammation and the inflammation is mediated through the transcription of several proinflammatory cytokines, chemokines and adhesion molecules. So, both transcriptional factors KLF2 and NF-κB are being associated with the similar cellular functions and their maintenance. It was shown that KLF2 regulates most of the NF-κB-mediated activities. In this review, we focused on emphasizing the involvement of KLF2 in health and disease states and how they interact with transcriptional master regulator NF-κB.

## 1. Introduction

Kruppel-like factor (*KLF*)s are the member of zinc finger family of DNA-binding transcription factors, which play diverse role in various biological processes including quiescence, proliferation, differentiation, development, growth and inflammation [1,2]. *KLF* family members are highly conserved with a total of 17 members (*KLF*1 to *KLF*17) reported so far. All members of this protein family contain three Cys_2_/His_2_ zinc finger motifs in tandem by which they bind to the common DNA-binding regions of transcriptional target sequences [3,4]. Among all the members, *KLF2* has been most widely studied for its role in lymphocyte biology, specifically for their survival, differentiation and trafficking. To determine its critical role in lymphocyte biology, it was shown that the *KLF2* deficient mice die in prenatal stage highlighting its crucial role in embryonic development. Besides lymphocyte development, it has been functionally associated with erythropoiesis, lung development, hemodynamic regulation, T-cell survival, migration and trafficking [1,2,5]. In addition to the lymphocyte biology, it has been shown that KLF2 plays an important role in regulating proinflammatory activation in endothelial cells as well as in monocytes [6,7]. Emerging evidences show that the role of KLF2 is not only limited to the immune cell function and regulation, KLF2 also plays critical regulatory role in some abnormal and pathological conditions. For example, KLF2 plays important role in regulating adipogenesis and inflammatory disease conditions, such as, rheumatoid arthritis, vascular diseases, chronic infections and various malignancies [8,9,10].

The function mediated by KLF2 is through negatively regulating inflammation and reducing proinflammatory activity of nuclear factor kappa B (NF-κB) [6]. Many published data established the role of NF-κB as a key regulator of proinflammatory signals in various inflammatory conditions as well as in cellular malignancies and reviewed in several articles [11,12]. In case of inflammation or external impinges like bacterial infection or stimulus, NF-κB signaling activates the first line of immune defense, the innate immune system. In this review, we will discuss about the role of KLF2 in NF-κB-mediated regulation of inflammation. Part of the molecular mechanisms show that the KLF2 inhibits the expression of proinflammatory signals by co-recruiting chromatin modulators p300/cyclic adenosine monophosphate response element binding protein (CBP)-associated factor (PCAF), a critical NF-κB coactivators. Additionally, NF-κB inhibits KLF2 expression through interrupting the binding of MADS box transcription enhancer factor 2 (MEF2) factors and access of histone deacetylase (HDAC) molecules to *KLF2* promoter. Thus, both KLF2 and NF-κB interplay between them in regulating inflammatory cascades. In this review, we also focused on unveiling the regulatory role of KLF2 in various relevant tissues and cells such as, lungs, T cells, T-regulatory (T-reg) cells, endothelial cells and monocytes that are associated with the various inflammatory and pathological conditions.

## 2. Kruppel-Like Factor

Kruppel-like factor (*KLF)* family members are evolutionary conserved and named based on their homology with *Drosophila* “Kruppel” protein and is derived from German word “cripple” [13]. In *Drosophila*, the *gap* gene encodes Kruppel protein, which is a zinc finger transcription factor, responsible for segmentation in embryonic stage and the mutation in the gene results in crippled appearance of larva. Apart from mammals, KLF proteins have homologs in *Gallus gallus* (chicken), *Daniorerio* (zebrafish), *Xenopuslaevis* (frog) and *Caenorhabditis elegans* (nematode). The members of the KLF transcription family exhibit a characteristic presence of conserved carboxyl terminus consisting of three Cys_2_-His_2_ zinc fingers containing DNA binding domains that bind to common CACCC elements in GC-rich regions of their target genes to regulate their activation and repression [3].

Erythroid Kruppel-like factor (EKLF) or KLF1 was first identified in mouse erythroleukemia cell line. It regulates the transcription of β-globin promoter gene and critically maintains the erthyropoiesis process [14]. There are now 17 known mammalian KLF proteins, with distinct N-terminal sequences and consist of various combinations of transactivation/repression domains [2].

The mammalian *KLF*s are categorized in three groups based on their shared domain architecture, common mechanisms of regulation, recruiting transcriptional regulatory proteins that include transcriptional coactivators and corepressors and other chromatin remodeling proteins as shown in Table 1. Based on phylogenetic analysis, *KLF*s 15 and 17 are more distantly related and harbor no defined and established protein interaction motifs. They vary widely in their expression pattern based on tissue/cell dependent manner and regulate divergent cellular processes.

## 3. Kruppel Like Factor 2

**Figure 1:**
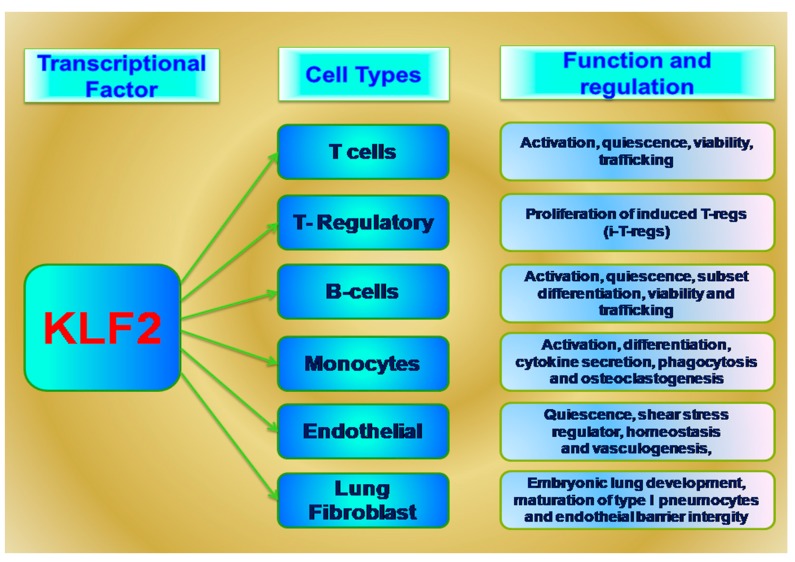
Kruppel-like factor 2 (KLF2) mediated functions in various cell types.

KLF2 has been most widely studied for its role in hematopoietic cell biology and inflammatory diseases shown in Figure 1. It is also known as lung Kruppel-like factor (LKLF) based on its first finding in lung tissues [5]. The human *KLF2* gene is located at chromosome 19p13.1 and is highly conserved between human and mouse homologs, with 85% nucleotide sequence identity and 90% amino acid similarity [2,15]. Interestingly, the *KLF2* homology present in two species with similar number of exons and introns in the transcriptional coding region and share similarity in 5′ and 3′ untranslated regions, suggesting that similar mechanisms regulate the expression of both homologs.

### 3.1. KLF2 in Lungs

Using the zinc finger domain of EKLF as a hybridization probe, *LKLF*/*KLF2* gene has been first discovered in mouse genomic library [16]. It is also expressed in heart, skeletal muscle, kidney, testis, lymphoid organs like thymus and spleen [5,16]. In mouse embryo, *KLF2* gene has been found to be developmentally regulated, with a peak expression at 7 days followed by a down-regulation at 11 days and further reactivation. It has been shown that *KLF2* knockout mice lack mature type I pneumocytes and die postnatally. Studies have shown that thyroid hormone and glucocorticoid receptors are important mediators in lung development and KLF2 is a necessary regulator for thyroid hormone and silencing mediator of retinoid and thyroid hormone receptors (SMRT)-mediated maturation of type I pneumocytes in the lung [17]. It has been also shown that lung KLF2 expression is significantly reduced in vivo rodent models on exposure to acute lung injury by influenza virus, lipopolysaccharides (LPS), or high-tidal-volume mechanical ventilation [18]. Endothelial barrier integrity is maintained by KLF2 and guanine nucleotide exchange factor 3 (RAPGEF3)-Ras-related C3 botulinum toxin substrate (RAC)1-mediated signaling. Mechanistically, KLF2 promotes Rac1 expression by transcriptional activation of RAPGEF3 through its multiple CACCC sites in the promoter and enhances endothelial barrier integrity in KLF2-expressing cells. However, the alteration in endothelial KLF2 leads to dysregulated lung microvascular homeostasis and pathological condition in lung tissue. Thus, KLF2 plays important role in lung development.

### 3.2. KLF2 in T Cells

KLF2 is expressed in single-positive CD4+ and CD8+ T cells and remains highly expressed in both naïve and memory T cells [5]. Its expression is rapidly lost after T cell receptor (TCR)-mediated activation of T cells. It has been shown that during T cell stimulation, both KLF2 mRNA and protein are down-regulated. KLF2 is expressed in thymocytes at the single positive stage and not seen during earlier double negative or double positive stages; whereas *KLF2*-null T cell spontaneously exhibits an activated phenotype. Thus, KLF2 regulates T cell homeostasis by promoting T-cell quiescence. Due to the prenatal death of *KLF*-null mice, to study the *KLF2*-mediated regulation, *KLF2*^−^/^−^*RAG*^−^/^−^ (recombination activating gene) chimeric mice were used [5]. KLF2 maintains the viability of T cells in the peripheral lymphoid organs and blood and mediates anti-apoptotic stage of mature single positive T cells. The induced KLF2 deficiency leads to death in the spleen and lymph nodes from Fas (CD95)-ligand mediated apoptosis of T cells [5].

Further study showed that the quiescent status of T cells is maintained through negatively regulated c-myelocytomatosis (Myc)/mitotic arrest deficient (Mad)-dependent pathways and by inducing expression of the cell cycle inhibitor p21^cip1^ [19]. Study has shown that KLF2 deficiency stimulates surface expression of co-stimulatory molecules CD40 and CD86 in dendritic cells and increases T cell activation [20]. In addition to maintaining cellular quiescence, KLF2 also plays a critical role in controlling T cell trafficking from thymus to secondary lymphoid tissues by directly regulating CD62L, an adhesion receptor vital for T cell egress from the blood into secondary lymphoid tissues and Sphingosine 1-phosphate receptor 1 (S1P_1_), a lysosphingo lipid receptor that regulates T cell trafficking from thymic and peripheral lymphoid tissue. The KLF2-null thymocyte shows impaired trafficking, whereas KLF2-transduced T cells showed increased accumulation in lymphoid organs [21].

It is apparent that the KLF2 is expressed in naïve T cells, down regulated in activated cells and again expressed in mature memory T cells. In general, T cells remain in a quiescent state until they encounter their cognate antigen bound to a major histocompatibility complex (MHC) molecule on the surface of a respective antigen-presenting cell. TCR activation leads to increased proteolysis of KLF2 protein through ubiquitination and reduced mRNA expression. KLF2 downregulation is a sequential response mediated through interaction of T-cell receptor (TCR) ligand and the integrated activation of protein kinase B along with the mitogen-activated protein kinase (MAPK) and extracellular signal-regulated kinase 1/2 (ERK 1/2) [22]. The presence of continuous TCR signaling and cytokines, are required for KLF2 degradation and in absence of these signaling KLF2 is re-expressed. In in vitro studies, reactivation of KLF2 in memory cells occurs in response to low dose of cytokines such as interleukin (IL): IL-2, IL-7 and IL-15, which induce CD8^+^ T cells to differentiate into memory T cells. In contrast, IL-4, IL-12 and low dose of IL-2 maintains effector T cells state and inhibits the re-expression of KLF2 [23]. Study showed that KLF2 expression inhibits the expression of inflammatory chemokine receptor C-X-C motif chemokine receptor (CXCR)3 in immune activated CD8^+^ T cells [22]. Further study revealed that partially blocking KLF2 down regulation with MEK inhibitor induces CXCR3 in CD8^+^ T cells. Therefore, loss of KLF2 in CD8^+^ T cells is required for expression of inflammatory chemokine receptor CXCR3 to facilitate binding with C-X-C motif chemokine 10 (CXCL10) for its clonal expansion.

### 3.3. KLF2 in T-Regulatory Cells

The transcription factor KLF2 has been shown to be a rate-limiting factor to increase the proliferation of regulatory T cells (T-regs) [24]. T-reg cells are subset of CD4^+^ T cells, vital to maintain self-tolerance and prevent autoimmunity by functionally suppressing auto-reactive lymphocytes. T-reg cells are positive for CD4, fork-head box P3 (FoxP3) and CD25 markers. T-reg cells development and function is mediated through FoxP3, which is transcriptionally regulated by KLF2 in S1P_1_-dependent manner. KLF2 directly and epigenetically regulates FoxP3 expression in induced T-reg cells (i-T-reg). Direct binding was proven through chromatin immunoprecipitation (ChIP) assays, which showed KLF2 binds in the FoxP3 promoter and enhancer sites. Mechanistically, acetylated histone H4 (H4Ac) marks designate transcriptionally permissive areas of chromatin and KLF2 helps in chromatin remodeling, which introduces more H4Ac marks in FoxP1 promoter and enhancer regions and leads to binding of signaling assembly including histone acetyltransferases that reverse PCAF mediated FoxP3 gene silencing. KLF2 has been found as a rate-limiting factor during this inductive process and the TCR-mediated KLF2 proteolysis limits i-T-reg production. It has been shown that Transforming growth factor-beta (TGF-β) mediated signaling event partially reduces TCR-mediated KLF2 degradation and increases i-T-reg production. The administration of drug Simvastatin (3-hydroxy-3-methylglutaryl coenzyme A reductase inhibitor) induced KLF2-mediated production of i-T-regs in cultured CD4^+^CD25^−^ T cells or thymocytes. However, the same effect could be seen through overexpression of KLF2 using an established mammalian vector, highlighting that FoxP3 expression is transcriptionally regulated by KLF2 and depends on KLF2 protein stability [24]. Thus, chemicals and drugs, which enhance KLF2 stability would be important for T-regs production and would be helpful in regulation of autoimmune diseases. However, in malignancies, T-reg cells help cancer cells to evade treatment response. Therefore, increased T-reg would predict poor outcomes. Limiting T-reg production by inducing KLF2 proteolysis via E3 ubiquitin ligases, such as WW domain-containing protein 1 (WWP1), Smad ubiquitination regulatory factor 1 (SMURF1), or F-box/WD repeat-containing protein 7 (FBW7) could be more effective in managing malignancies [25,26,27].

## 4. KLF2 in B Cells

KLF2 expression is also present in B cells. Similar to the association with T cells, KLF2 acts as a quiescence factor for B cells. Its expression decreases upon B cell activation and again re-expressed in plasma and memory B-cells [28,29]. In mice, depending upon differences in tissue distribution, trafficking potential and function, three subsets of B-cells exists: follicular (FO), marginal zone (MZ) and B1 B cells [30]. It has been shown that KLF2 is differentially expressed among the mature B cell subsets in the periphery, with highest expression seen in B1 B cells, comparatively lower expression in FO zone and least in MZ cells [31]. Hoek et al. showed that KLF2 deficiency results in immature B cell retention within bone marrow [32]. In vivo deficiency of KLF2 had no substantial effect on later stage B cell development and total number of B cells remains unchanged [31]. However, it regulates subset differentiation and maintenance of mature peripheral B-cells. In in vivo models, its deficiency results in increased population of MZ cells and decreased B1 B cells [31,32]. KLF2 is essential for proper trafficking of B cell subsets. It induces decreased expression of trafficking molecules and chemokine receptors, such as CD62L, β7-integrin, CXCR5 and S1P_1_ and results in altered B cell tissue distribution, with increased B cells seen in spleen and comparatively reduced number in blood and spleen [31,32]. It also differentially regulates expression of migratory receptors and specifically promotes or suppresses chemokine specific migration in MZ and FO B cells, respectively [32]. Its disruption leads to lower level of CXCR5 and S1P_1_ in MZ cells and increased levels of these factors in FO cells. KLF2 deficient FO B cells showed induced chemokine-mediated migration compared to MZ cells and abrupt anatomical repositioning within the spleen [32]. Instead of its critical role in B cell trafficking, KLF2 deficiency does not affect ex vivo humoral immune responses [32]. However, KLF2 deficient B cells exhibit reduced capacity to respond to anti-IgM stimulation. Forced KLF2 expression inhibits pre-B cell receptor (BCR)-mediated proliferation, by upregulation of cell cycle inhibitors p21 and p27 and downregulation of c-myc [33]. Further study has shown that KLF2 over expression reduces the expression of activation molecules in LPS induced B cells and impairs B cell activation. KLF2 overexpression also induces apoptosis in LPS-stimulated splenic B cells [33]. Thus, KLF2 plays critical role in quiescence, trafficking, function and terminal differentiation of B cells. 

### 4.1. KLF2 in Endothelial Cells

In endothelial cells, KLF2 acts as a shear stress inducible transcription factor, with site and level of expression corresponding to the shear forces in vascular development and shear stress to blood flow in adult epithelium [34]. KLF2 has been shown as a master regulator of endothelial cell quiescence. It critically regulates the expression of endothelial genes during vasculogenesis and also maintains the health of vascular endothelium in response to shear stress due to flow [35]. Further, KLF2 plays vital role in endothelial cells, as its deletion leads to embryonic lethality, vascular complication such as change in vascular tone, induces bleeding and also cardiac dysfunction [15]. 

The expression of adhesion molecules such as vascular cell adhesion molecule (VCAM)-1 and E-selectin have been shown as initial events in endothelial cells against inflammatory response and induces leukocyte attachment and rolling on the endothelial surface [36,37]. In cultured human umbilical endothelial cells, KLF2 is induced by laminar shear stress and inhibited by proinflammatory cytokines. The overexpression of KLF2 has been shown to highly induce thrombomodulin and endothelial nitric oxide synthase (eNOS) expression and strongly inhibits the expression of proadhesive/procoagulant factors, such as VCAM-1 and tissue factor. Thus, KLF2 strongly attenuates the expression of cellular adhesion molecules in endothelial cells.

The integrity of endothelial monolayer and the endothelial barrier function serves as first layer of protection against infectious agents, mechanical factors and chemicals. Intercellular junctions and cytoskeletal remodeling critically regulate endothelial barrier function. These are further modulated by the activities of small guanosine triphosphate (GTP)ases, particularly the Rho family of GTPases, such as the Ras homolog gene family member A (*RhoA*), cell division cycle 42 (Cdc42) and Rac1 and transcriptionally regulated by KLF2 as shown in case of acute lung injuries in rodent models [18]. 

A recent study showed that atheroprotective biomechanical stimulus elicited by laminar shear stress inhibits endothelial cell metabolism via KLF2-mediated repression of 6-phosphofructo-2-kinase/fructose-2,6-biphosphatase-3 (PFKFB3) [38]. They showed that laminar shear stress reduced glucose uptake and mitochondrial content in endothelial cells. Interestingly, the reduction in metabolism was rescued by silencing *KLF2* gene. The inhibitory effect of KLF2 on glucose metabolism was also observed in in vivo mice models. KLF2 knock down in mice showed normal glucose uptake in endothelial cells of perfused hearts, whereas, the over expression of KLF2 has the inhibitory effects on endothelial glycolysis induced by laminar flow. Mechanistically, KLF2 represses PFKFB3 promoter activity and ultimately affects glycolysis process. Moreover, the induced overexpression PFKFB3 was shown to be sufficient to reverse KLF2-mediated reduction in angiogenesis and endothelial cells network formation. Thus, this study highlights the involvement of KLF2 and glycolytic enzyme in regulation of endothelial cell phenotype under laminar shear stress.

The expression of inflammatory and adhesion molecules in endothelial cells are modulated by interaction with adjacent smooth muscle cells. Specifically, endothelial cells when cultured with a direct contact of quiescent smooth muscle cells in presence of TNF-α, showed remarkably increased level of KLF2 expression, concomitantly decreased amount of NF-κB nuclear translocation and reduced expression level of adhesion molecules such as intercellular adhesion molecule (ICAM)1 and VCAM1 [39]. However, the expression of KLF2 in mouse embryonic fibroblasts and in endothelial cells is negatively regulated by adaptor protein p66shc through limiting MEF2A promoter binding independent of NF-κB expression [40]. Thus, KLF2 is an important transcriptional regulator for the inflammatory response and expression of adhesion molecules in endothelial cells and mediates the intercellular signals under various pathophysiological conditions.

### 4.2. KLF2 in Monocytes

Monocytes are important component of innate immune system and are involved in cellular defense mechanism. Under normal physiological conditions they remain in an inactive state or quiescence condition. Upon injury or after induction with proinflammatory stimuli such as cytokines/chemokines, they differentiate into macrophages and regulate tissue injury and repair process. KLF2 has been identified as a novel regulator of proinflammatory factors in monocytes [6]. KLF2 expression in monocytes has been first shown and functionally studied by our group. Primary human monocytes show robust expression of KLF2 compared to the human monocytic cell line, which shows reduced expression. KLF2 expression in monocytes is also reduced with activation, differentiation into macrophages and osteoclastogenesis in pathological conditions [6,8,41]. The induced overexpression of KLF2 attenuated LPS-mediated activation of monocytes and reduced biological function, such as phagocytosis [6]. Interestingly, its forced expression sufficiently reduced the inflammatory effects both in in vitro and in vivo models [6]. Thus, KLF2 has been identified as an important modulator of proinflammatory signals. In murine model, KLF2 overexpression does not inhibit but rather augments monocytic recruitment to an inflammatory site, however, it inhibits the expression of cytokines and chemokines [6]. Thus, the anti-inflammatory effect of KLF2 is likely due to inhibition of proinflammatory gene expression in monocyte and not recruitment. Similar to T-cells, in monocytes, the KLF2 expression reduces with activation and differentiation into macrophages [6]. Thus, reduction in KLF2 expression is similar to that associated with T cells after activation [5]. It has been found that in monocytic cells, the anti-inflammatory effects of KLF2 are mediated through NF-κB, AP1 and several coactivator molecules [6]. The KLF2-mediated mechanistic regulation of anti-inflammatory effects in monocytes has been of continuous research interest in inflammatory diseases. 

Monocyte activation is prerequisite for normal immune response, however this process must be tightly controlled to prevent host tissue damage and autoimmune disease. The deregulated and prolonged activation of monocytes has deleterious effects in several inflammatory diseases and chronic infectious conditions such as rheumatoid arthritis, artherosclerosis and sepsis [8,42,43,44]. Due to the role of KLF2 in regulation of monocytes activation, it has been further studied in these inflammatory diseases. Interestingly, in in vitro and in vivo disease models, KLF2 deficiency induces expression of proinflammatory cytokines expression, leads to abrupt monocyte activation/differentiation, osteoclastogenesis and mediated aggressive disease symptoms and has been of further extensive research interest, as discussed in detail in the disease section. 

## 5. Inflammation Master Regulator NF-κB

NF-κB transcription factor has been well known for its central role in regulation of inflammation [11]. Role of NF-κB has been widely studied in various inflammatory diseases [45]. KLF2 has been identified as an inhibitor of NF-κB mediated inflammatory activities and hence has been of research interest in inflammation and mediated chronic inflammatory diseases [6,7,42,44]. These two transcription factors tightly regulate the expression of various proinflammatory cytokines and affect the cellular inflammatory responses. In this section, we present a brief overview of NF-κB transcription factor, its regulation and cross talk with KLF2 in inflammatory regulation. 

## 6. NF-κB Protein Family

The NF-κB family of proteins is composed of two subfamilies: NF-κB and reticuloendotheliosis (Rel) [46]. All the proteins of NF-κB family share a conserved 300-amino acid region, DNA-binding/dimerization domain known as Rel homology domain (RHD) in their NH_2_-terminus [47,48]. 

The Rel subfamily proteins include: RelA, RelB and c-Rel and they consist of the transactivation domain in C-terminal region. The NF-κB family includes two proteins NF-κB1 and NF-κB2, which are synthesized from large precursor proteins p105 and p100, respectively. They contain long C-terminal domains with multiple copies of ankyrin repeats, which act to inhibit these proteins. The p105 and p100 undergoes ubiquitin/proteasome pathway-mediated selective proteolysis of their C-terminal region containing ankyrin repeats, leading to generation of active forms p52 from p100 and p50 from p105. These smaller p50 and p52 proteins, contains no intrinsic ability to activate transcription and are proposed to act as transcriptional repressors when binding κB elements as homodimers, except when they form heterodimers with Rel family. A brief summary of NF-κB family protein is shown in Table 2.

## 7. Structure, Function and Regulation of NF-κB

NF-κB protein acts as rapid acting transcription factor, regulating various cellular responses. It binds to the enhancer or promoter regions of several genes with variations of consensus DNA sequence 5′-GGGRNWYYCC-3′ (where N = any base; R = purine; W = adenine or thymine; Y = pyrimidine), known as κB sites. It remains in an inactive state due to binding with inhibitory IκB proteins and remains in the cytoplasm. The interaction with IκB protein interrupts the nuclear localization of NF-κB dimer and interferes with DNA binding and transcriptional activator role [49]. There are several inhibitory IκB proteins (IκBα, IκBβ, IκBγ and IκBε) with differential affinities to NF-κB dimers [50]. The inducers of NF-κB signaling include proinflammatory cytokine, TNF-α, IL1-β, LPS, DNA damaging chemicals and ionization radiation [51,52]. These inducers are recognized by the receptor on the surface of cells or by one inside the cells. Receptor activator of NF-κB (RANK) is a type of TNF receptor and is central activator of NF-κB [53].

The activation of NF-κB involves various canonical and non-canonical mechanisms [46]. However, till date the mechanism of activation is not fully understood. Overall, the inducer binding activates inhibitor of κB kinase (IKK) complex, which mediates phosphorylation, poly-ubiquitination and proteasome-mediated degradation of inhibitory IκB proteins. The activation of NF-κB also involves various covalent modifications of NF-κB [54]. The release of IκB from heterodimers activates nuclear localization of active NF-κB proteins, leads to binding to κB sites and induces inflammatory response. The NF-κB-dependent gene transcription involves the recruitment of RelA/p65 subunit to cognate genomic κB sites and requires transcriptional co-activators that induce the sequence-specific activators and leads to altered chromatin structure and induces transcriptional activation. One of the known coactivator of RelA/p65 subunit is CBP-binding protein and its structural homolog p300 [55]. The protein kinase A (PKA), phosphorylates p65 subunit and enhances its association with PCAF. PCAF is a member of histone acetyltransferase family and induces acetylation, alters chromatin and results in transcriptional induction. Enriched histone acetylation at promoters of proinflammatory cytokines (such as IL-1, IL-2, IL-8 and IL-12) has been found with increased transcriptional activation of these gene and resulting inflammation [56].

The effect of NF-κB activation is found to be transient, with nuclear form remaining active for less than an hour. The activated NF-κB also induces the transcription of genes coding IκB proteins and results in a feedback loop of inhibition by IκB proteins [57]. The newly synthesized IκB moves to the nucleus and complexes with NF-κB heterodimer (p65-p50), interrupts the binding with κB sites and the p65-p50-IκB complex moves to the cytoplasm. This, results in resolution of activated inflammatory response. In case of constitutive presence of induction signal, the NF-κB oscillates from latent to active form. The key role of this oscillation still remains uncertain. The transient activation of NF-κB and its oscillation to latent form insures that induction of inflammatory responses is tightly regulated and occurs only in response to external stimulus.

In multicellular organisms, NF-κB plays central role in innate response as the first line of defense to external environmental danger and also regulates adaptive immune response found in higher vertebrates [58]. In addition to regulating immune response and inflammation, NF-κB induces anti-apoptotic signal to secure the healthy cells, which might be killed in case of inflammatory response against infectious invaders. Therefore, the feedback inhibitory loop and resolution of the response induced by NF-κB activation is a critical step. The constitutive anti apoptotic signal can also lead to build up of otherwise dangerous cells and may lead to several pathologic conditions, including cancer. The evolutionary relationship and opposing functions of NF-κB in inflammation and cancer was reviewed in length [11]. Elaborate regulatory pathways involving various genetic/mutational events, which result in deregulated NF-κB pathway in several human genetic diseases, are summarized [12].

## 8. KLF2-Mediated Regulation of NF-κB Transcriptional Activity and Function in Monocytes

**Figure 2:**
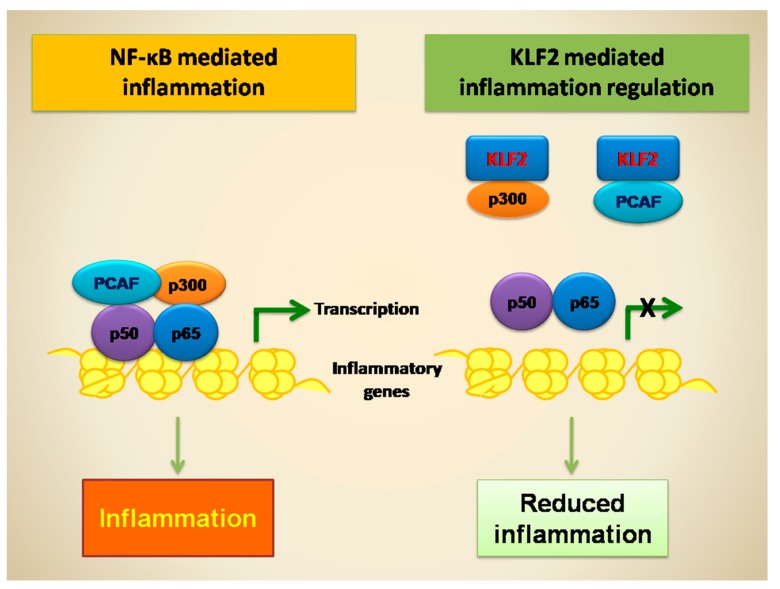
NF-κB and KLF2 in transcriptional regulation. PCAF: p300/cyclic adenosine monophosphate response element binding protein (CBP)-associated factor.

In monocyte, it has been shown that KLF2 overexpression significantly inhibits the expression of several cytokines/chemokines (IL-1, IL-8, TNF, monocyte chemoattractant protein (MCP)-1, macrophage migration inhibitory factor (MIF), macrophage inflammatory protein (MIP) and CD40L) and inflammatory factors (tissue factor and cyclooxygenase (COX)-2) [6,8,44]. Most of these have been identified as downstream targets of NF-κB. However, the regulation of these factors by KLF2 is only partially understood. In vitro studies showed that KLF2 does not alter the nuclear accumulation of p65 [6]. It either does not affect the phosphorylation of IκB or its degradation; or the localization of kinases IKKα, IKKβ and IKKγ; or the NF-κB binding. However, KLF2 strongly inhibits p65-mediated transactivation of NF-κB concatemers in monocytes that were co-transfected with KLF2 expressing and NF-κB luciferase reporter plasmid [6,44]. Thus, KLF2 inhibits NF-κB transcriptional activity and has been identified as important target to regulate chronic and acute inflammation [6,44]. Mechanistically, KLF2 inhibits NF-κB transcriptional activity by directly interacting with p300 and PCAF, which are critical co-activators for NF-κB-mediated transcriptional activity [6,7,42] (see Figure 2). KLF2 reduces the coactivators recruitment to NF-κB and also attenuates the histone 3 lysine 9 (H3K9) and H4K8 acetylations. However, the co-transfection experiments, with exogenous PCAF significantly rescues the KLF2 mediated inhibition of NF-κB transcriptional activity [6]. Thus, KLF2 regulates p65 transcriptional activity by counteracting with p300 and PCAF and reduced H3K9 and H4K8 acetylation.

## 9. KLF2-Mediated Regulation of NF-κB Transcriptional Activity and Function in Endothelial Cells

The transcriptional activity of NF-κB and KLF2 activity is antagonistically regulated in human umbilical vein endothelial cells [59]. In endothelial cells, the induction of proinflammatory cytokines reduces the expression of KLF2. Interestingly, in presence TNF-α stimulation, NF-κB inhibits the expression of KLF2. This TNF-α-mediated inhibition of KLF2 is dependent on p65 expression and occurs through a 107-bp region (−221 to −114) of the KLF2 promoter and is independent of binding to an NF-κB binding site. It was shown that p65 interacts with HDAC4 and HDAC5 and interrupts the binding of MEF2 to induce the KLF2 promoter activity [59]. This was the first evidence showing NF-κB-dependent epigenetic regulation of KLF2 by HDACs. Thus, in normal cellular conditions the inter-balance between KLF2 and p65 activity might regulate the cellular activation. ChIP and luciferase promoter assays showed that HDAC5 directly interacts with KLF2 promoter and reduces its transcriptional expression. Silencing of *HDAC5* leads to increase in *KLF2*-dependent induction of *eNOS* gene expression [60]. Further studies also highlighted the epigenetic regulation of KLF2 mediated by HDAC4 and HDAC5. In mouse fibroblasts, under serum starvation, KLF2 transcription increases, which decreases the random cell motility through glycogen synthase kinase (GSK)-3β-mediated proteosomal degradation of HDAC4 molecule [61]. These studies suggest that KLF2 expression is epigenetically regulated and involves multiple upstream signals.

## 10. KLF2 in Inflammatory Diseases

KLF2 plays a key role in regulating inflammation. It has been shown as a novel transcriptional regulator of proinflammatory activation in vascular endothelial cells and monocytes [6,7]. The vascular endothelium acts as interface between blood and tissues and is involved in the biological response to inflammation. Proinflammatory stimuli such as cytokines and hemodynamic stress affect endothelial cell dysfunction and induce pro-adhesive and pro-thrombic phenotype [62,63]. These events mediate important adaptive responses. In addition, monocyte activation is prerequisite for normal innate immune response against various infectious conditions and the deregulation of this event results in host cell damage and pathological conditions. The process of inflammation is characterized by monocyte activation and recruitment to of macrophage to site of infection/injury. Thus, dysregulated activation of endothelial cells and monocytes results in development of vascular disease states resulting in several chronic inflammatory diseases such as, atherosclerosis, rheumatoid arthritis, inflammatory bowel diseases, and sepsis [6,8,9,42,43,63,64].

**Figure 3:**
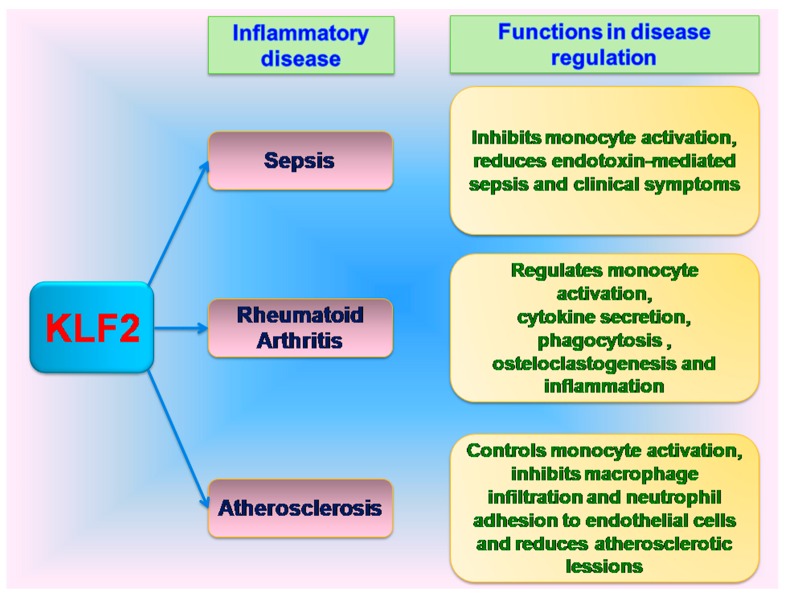
Role of KLF2 in inflammatory disease regulation.

As discussed earlier, NF-κB plays a central role in inflammatory response and also regulates the resolution phase, in a negative feedback manner after the removal of foreign agent. However, the deregulated NF-κB pathway also leads to chronic inflammatory conditions. Inhibition of NF-κB pathway with anticytokine therapy showed positive effects in regulating inflammatory conditions [65,66]. Due to the anti-inflammatory effects of KLF2 on myeloid cell activation, negative regulation of cytokines secretion and NF-κB activity; it has been further studied for possible role in regulation of chronic inflammatory diseases and infectious conditions. Interestingly, a reduction of 30–50% KLF2 has been observed in human subjects with acute or chronic inflammatory conditions [6,42]. Further, studies have been shown the role of KLF in regulation chronic infections and inflammatory disease such as sepsis, rheumatoid arthritis, atherosclerosis (Figure 3).

## 11. KLF2 in Sepsis

Role of KLF2 has been shown in sepsis, which is a chronic bacterial infection. In vitro KLF2 deficiency induces LPS-mediated sepsis symptoms [44]. It has been shown that KLF2 and hypoxia induced factor-1 alpha (HIF-1α) signaling plays a role in inflammatory regulation induced by Gram-positive endotoxin-mediated sepsis [10]. In primary macrophage cells and macrophage cell lines, inoculation of live or heat-inactivated Gram-positive bacteria or Gram-positive endotoxin alone induce *HIF-1α* mRNA and protein expression. Under same conditions, Gram-positive endotoxin reduces the expression of anti-inflammatory transcription factor, KLF2. Further, it has been shown that KLF2 regulates HIF-1α expression and its silencing increases *HIF-1α* mRNA and protein expression. Interestingly, endotoxins from Gram-positive bacteria also reduce KLF2 expression and induce the HIF-1α expression, in NF-κB-dependent manner [42]. The *KLF2* deficient mice have more severe Gram-positive endotoxin-mediated sepsis and clinical symptoms. In contrast, *HIF-1α* deficient mice induced protection against endotoxins. Thus, in addition to the known effects on inhibition of proinflammatory gene expression in myeloid cells, KLF2 in association with HIF-1α could efficiently inhibit the Gram-positive endotoxin-induced effects in sepsis and can efficiently reduce clinical symptoms. Thus, KLF2 has been identified as a potent inhibitor of NF-κB dependent HIF-1α signaling and is a critical determinant of myeloid cell activation and host response in polymicrobial infection and endoxemia.

## 12. KLF2 in Rheumatoid Arthritis

Due to the role of KLF2 as a negative regulator of monocyte activation and function, we focused on the role of KLF2 in rheumatoid arthritis, which is a chronic inflammatory disease, with severe inflammation and destruction of synovial joints in bones and ligaments [67]. Apart from antigen-dependent activation by T cells, it involves the recruitment of B-cells and monocytes to the inflammatory site, increased secretion of proinflammatory cytokines and differentiation into tissue macrophages [68,69]. The macrophage differentiated into osteoclasts mainly mediates subchondrial bone destruction in rheumatoid arthritis [41]. The first evidence of inflammatory regulator role of KLF2 was seen in mice model, where, overexpressed of KLF2 effectively inhibits the paw edema induced by Carrageenan [6]. In monocytes, under in vitro conditions, KLF2 inhibits secretion of cytokines/chemokines, strongly inhibits the differentiation/activation of monocytes and also inhibits phagocytosis. Thus, by inhibiting these inflammatory stimuli KLF2 reduces the autocrine/paracrine activation of surrounding immune and non-immune cells and modulates the inflammation and paw edema after carrageenan administration. Further, due to chronic inflammatory condition found in rheumatoid arthritis, we explored the involvement of KLF2 in pathogenesis and possible regulation of rheumatoid arthritis. Using *KLF2* hemizygous mice we studied its role in methylated bovine serum albumin (mBSA) and IL-1β-induced arthritis conditions [8]. In parallel, with the role of KLF2 in monocytes, the *KLF2* hemizygous mice showed increased induction of inflammatory genes *MCP-1*, *COX-2* and plasminogen activator inhibitor-1 (*PAI-1*) in bone marrow derived monocytes compared to wild type control mice. Interestingly, the *KLF2* hemizygous mice showed increased recruitment of inflammatory CD11b^+^F4/80^+^Ly6C^+^ monocytes in peripheral blood and peritoneum and showed severe arthritic damage in cartilage and bones. The recruitment of monocytes and macrophage was increased in *KLF2* hemizygous arthritic bone tissues and was shown by higher number of triiodothyronine receptor auxiliary protein (TRAP) positive osteoclasts compared to wild type arthritic control mice. It was also shown that *KLF2* hemizygous mice harbored significantly higher osteoclastic differentiation, with increased number of nuclei, compared to wild type controls. These finding indicates that hemizygous *KLF2* mice represented severe disease condition under induced arthritis. Therefore, it further validates the role of KLF2 in controlling in inflammation. This finding further corroborates the earlier observation of decreased inflammation with KLF2 overexpression in carrageenan-induced inflammation in severe immune compromised mice [6]. Due to the known roles of NF-κB in rheumatoid arthritis [45] and the initial findings of anti-inflammatory effects of KLF2 in induced arthritic models, we hypothesized that KLF2 possibly reduce arthritis by negatively regulating NF-κB via the recruitment of PCAF [6]. The *KLF2* hemizygous arthritic mice also showed higher level of matrix degrading enzymes, matrix metalloproteinases (MMPs), such as MMP9 and MMP13, which activates other proteases and mediates a cascade of matrix degradation process resulting in the destruction of bones, cartilages and articular structures in rheumatoid arthritis [66]. It has been shown that KLF2 is an important inflammatory inhibitor of several cytokines such as tumor necrosis factor-alpha (TNF-α), IL-1β, MCP-1, MIP (macrophage inflammatory proteins)-1α, and IL-8. In addition, it inhibits the monocytes phagocytic activities and osteoclast differentiation. Therefore, these findings corroborated together suggesting KLF2 as a potential therapeutic target for rheumatoid arthritis treatment.

## 13. KLF2 in Atherosclerosis

Due to the important role in endothelial cells quiescence and vascular development, KLF2 has been further investigated for a possible protective role in the vascular system under pathological conditions [43]. KLF2 expression was significantly decreased in monocytes of the patients with atherosclerosis, a chronic low-grade inflammatory disease, characterized by higher expression of FOS (osteosarcoma gene) [6]. This observation indicated atheroprotective function of KLF2 and was established by using hemizygous *KLF2* deficient mice. It has been shown that KLF2 deficiency increased diet-induced atherosclerosis in apolipoprotein E-deficient mice [70]. Further study showed that KLF2 deficiency induces increased macrophage infiltration and neutrophil adhesion to endothelial cells and promotes atherosclerotic lesion in *KLF2* hemizygous mice [43]. Thus, atherosclerosis is associated with oxidative stress induced by imbalanced recruitment of monocytes and neutrophils to the impaired tissues. Increasing KLF2 level could be helpful in increasing protective functional interest in potential treatment for atherosclerosis.

## 14. Conclusions

KLF2 is an important transcriptional factor playing critical role in activation of various immune cells. It maintains the quiescence of T cells, endothelial cells and also inhibits the activation of monocytes [5,6]. The reduction in KLF2 levels observed with monocytes differentiation/activation is similar to the reduction in KLF2 after T cell activation and strongly supports a central role for KLF2 in the regulation of immune cell activation. KLF2 also plays key role in production of T-reg cells and can be modulated for sustained T-regs production.

Interestingly, NF-κB and KLF2 both acts as an antagonistic regulator of inflammation. In monocytes, KLF2 reduces the expression of several inflammatory genes and cytokines by regulating the transcriptional activity of NF-κB through competitive interaction with PCAF [6]. In endothelial cells, the KLF2 expression is regulated by MEF2 and HDACs, in p65 and TNF-α-dependent manner [59]. Thus, they are critical regulator of inflammatory balance under normal physiological condition. The KLF2 deficiency has been associated with chronic inflammatory diseases such as atherosclerosis, rheumatoid arthritis, Gram-positive endotoxin-induced sepsis [6,8,10,70].

In addition to regulation of myeloid cell activation and various inflammatory diseases, role of KLF2 is also emerging as a potential tumor suppressor gene owing to its roles in the inhibition of proliferation, migration and angiogenesis and in the induction of apoptosis, senescence and adhesion [71,72]. Several studies also further highlighted the role of KLF2 as an important factor to sustain embryonic stem cell ground state pluripotency, identity and self-renewal [73,74]. Thus, KLF2 could be novel promising target for treatment of inflammatory diseases, chronic infections, malignancies and also stem cell regulation and differentiation.

## 15. Future Directions

Due to the functional role of KLF2 as an anti-inflammatory and anti-atherosclerotic transcription factor, it may establish as a novel therapeutic target for various inflammation associated diseases and chronic infectious conditions. Any factors or molecules, which could stabilize KLF2 protein level, would be promising for treatment of inflammatory diseases (such as RA, atherosclerosis, sepsis, etc.) and also in the expansion of T-reg populations (for treatment of autoimmune diseases). However, in malignancies T-regs oppose to the desired immune responses. Hence, finding molecules that promote KLF2 degradation in T-cell compartment via ubiquitination or other processes would be of considerable interest. Future studies are needed to elucidate the detailed mechanistic regulation of KLF2 and NF-κB, which would help to better understand the cellular activation/differentiation pathways to develop potential targeted therapeutics for various inflammatory diseases.

## Figures and Tables

**Table 1:** Kruppel-like factor (KLF) family members.

KLF Group	Characteristics	KLF Family Members	Function
Group 1	Presence of CtBP- binding sites	KLFs 3, 8 and 12	Transcriptional repressors through their interaction with the carboxy-terminal binding protein (CtBP)
Group 2	Ability to bind deacetylases	KLFs 1, 2, 4, 5, 6 and 7	Transcriptional activators
Group 3	Presence of a Sin3A-binding sites	KLFs 9, 10, 11, 13, 14 and 16	Repressor activity through their interaction with the common transcriptional co-repressor Sin3A

**Table 2:** NF-κB subfamily.

Subfamily	Protein	Precursors	Gene
NF-κB	NF-κB1	p105→p50	*NFKB1*
	NF-κB2	p100→p52	*NFKB2*
Rel	RelA	p65	*RELA*
	RelB		*RELB*
	c-Rel		*REL*

## Abbreviations

KLF2	Kruppel-like factor 2
NF-κB	Nuclear factor-κB
PCAF	p300/cyclic adenosine monophosphate response element binding protein (CBP)-associated factor
MEF2	MADS box transcription enhancer factor 2
HDAC	Histone deacetylases
EKLF	Erythroid Kruppel-like factor
CtBP	Carboxy-terminal binding protein
LKLF	Lung Kruppel-like factor
SMRT	Silencing mediator of retinoid and thyroid hormone receptors
LPS	Lipopolysaccharides
RAPGEF3	Guanine nucleotide exchange factor 3
RAC	Ras-related C3 botulinum toxin substrate
RAG	Recombination activating gene
Myc	Myelocytomatosis
Mad	Mitotic arrest deficient
Cip	Cyclin-dependent kinase interacting protein
MHC	Major histocompatibility complex
S1P_1_	Sphingosine 1-phosphate receptor 1
TCR	T cell receptor
MAPK	Mitogen-activated protein kinase
ERK	Extracellular signal-regulated kinase
IL	Interleukin
CXCR	C-X-C motif chemokine receptor
CXCL10	C-X-C motif chemokine 10
T-regs	Regulatory T cells
FoxP3	Forkhead box P3
i-T-regs	Induced T-regulatory cells
ChIP	Chromatin immunoprecipitation
H4Ac	Acetylated histone H4
TGF-β	Transforming growth factor-beta
WWP1	WW domain-containing protein 1
SMURF1	Smad ubiquitination regulatory factor 1
FBW7	F-box/WD repeat-containing protein 7
FO	Follicular
MZ	Marginal zone
BCR	B cell receptor
VCAM	Vascular cell adhesion molecule
eNOS	Endothelial nitric oxide synthase
GTP	Guanosine triphosphate
RhoA	Ras homolog gene family member A
Cdc42	Cell division cycle 42
PFKFB3	6-phosphofructo-2-kinase/fructose-2,6-biphosphatase-3
ICAM	Intercellular adhesion molecule
Rel	Reticuloendotheliosis
IKK	Inhibitor of κB kinase
MCP-1	Monocyte chemoattractant protein-1
COX-2	Cyclooxygenase-2
GSK	Glycogen synthase kinase
HIF-1α	Hypoxia induced factor-1α
mBSA	Methylated bovine serum albumin
PAI-1	Plasminogen activator inhibitor-1
TRAP	Triiodothyronine receptor auxiliary protein
MMP	Matrix metalloproteinases
TNF-α	Tumor necrosis factor-α
MIP	Macrophage inflammatory proteins
RHD	Rel homology domain
RANK	Receptor activator of NF-κB
CBP	Cyclic AMP response element binding protein (CREB)-binding protein
PKA	Protein kinase A
MIF	Macrophage migration inhibitory factor
H3K9	Histone 3 lysine 9
